# Genome editing for blood disorders: state of the art and recent advances

**DOI:** 10.1042/ETLS20180147

**Published:** 2019-04-09

**Authors:** Marianna Romito, Rajeev Rai, Adrian J. Thrasher, Alessia Cavazza

**Affiliations:** 1 Infection, Immunity and Inflammation Program, Great Ormond Street Institute of Child Health, University College London, 30 Guilford Street, London WC1N 1EH, U.K.; 2 NIHR Great Ormond Street Hospital Biomedical Research Centre, London, U.K.

**Keywords:** blood disorders, genome editing, haematopoietic stem cells

## Abstract

In recent years, tremendous advances have been made in the use of gene editing to precisely engineer the genome. This technology relies on the activity of a wide range of nuclease platforms — such as zinc-finger nucleases, transcription activator-like effector nucleases, and the CRISPR–Cas system — that can cleave and repair specific DNA regions, providing a unique and flexible tool to study gene function and correct disease-causing mutations. Preclinical studies using gene editing to tackle genetic and infectious diseases have highlighted the therapeutic potential of this technology. This review summarizes the progresses made towards the development of gene editing tools for the treatment of haematological disorders and the hurdles that need to be overcome to achieve clinical success.

## Introduction

Genome engineering has endowed the scientific community with the ability to artificially modify genetic information, unlocking the potential of traditional medicine to new therapeutic approaches. Gene editing represents a platform in which programmable DNA nucleases specifically recognize a target genomic sequence where they introduce permanent genetic modifications. Four major classes of targeted nucleases have been described so far: meganucleases, zinc-finger nucleases (ZFNs), transcription activator-like effector nucleases (TALENs), and the CRISPR/Cas9 system (reviewed in [1]). These nucleases are designed to create double-strand breaks (DSBs) into target DNA sequences, triggering the activation of two major endogenous cellular repair mechanisms: non-homologous end joining (NHEJ) and homologous recombination (HR) [2]. NHEJ is efficient but error-prone, thus repair of the break site results in small DNA insertions or deletions (indels) [3]. Indels can disrupt target genes by shifting the reading frame or introducing stop codons, with consequent production of non-functional proteins. In contrast, HR results in accurate repair through the use of an undamaged DNA template with homology to the sequence flanking the DSB. Each of these pathways — NHEJ or HR — could potentially be exploited for therapeutic purposes. CRISPR/Cas9-based genome editing has been widely used in proof-of-principle preclinical studies holding great promise for further clinical applications. This review will focus on the application of precise editing to treat monogenic disorders affecting the haematopoietic system and on the factors that will determine its success in the clinical setting.

## Therapeutic genome editing

For many haematological conditions, such as primary immunodeficiencies and haemoglobinopathies, the only curative treatment is histocompatibility leukocyte antigen (HLA)-matched allogeneic haematopoietic stem cell transplantation (HSCT), which replaces defective haematopoietic lineages with functional cells. The limitations of this procedure include donor availability with the consequent risk of graft rejection, incomplete immune reconstitution, graft-versus-host disease, death and/or long-term dysfunction arising from the conditioning regimen [4,5]. Transplantation of autologous, genetically modified stem cells could represent an alternative to allogeneic HSCT, and many groups have worked over the last few decades toward achieving efficient and safe gene transfer to haematopoietic stem cells (HSCs). In order for gene therapy to be a viable and potentially life-long treatment, it is necessary to (1) correct a sufficient amount of long-term repopulating HSCs, (2) achieve a stable and regulated expression of the therapeutic gene, and (3) ensure that the process is safe. Pioneering gene therapy approaches for severe combined immunodeficiency disorders (SCID) using retroviral vectors have demonstrated the applicability of the technology to treat rare genetic diseases affecting the haematopoietic system [6]. However, viral vectors carry a potential risk of genotoxicity due to their semi-random integration pattern and to unregulated transgene expression in target cells [7,8].Using autologous genetically modified haematopoietic stem and progenitor cells (HSPCs), gene editing could represent an alternative to conventional gene therapy and overcome some of its limitations. Engineered endonucleases that introduce DSBs at specific sequences in the genome offer much more control over viral vector site integration; moreover, the site-specific insertion of DNA or correction of a disease-causing mutation *in situ* guarantees that physiologically regulated gene expression is preserved. There are different gene editing applications based on the two main repair mechanisms utilized by the target cells to correct the DSB. HDR (homology-directed repair) can be used to either insert a gene into a specific ‘safe harbour’, into its own locus or to specifically repair small or point mutations in the defective gene. In contrast, if NHEJ takes place, the generated indels could abolish the expression of the protein, or the function of a regulatory region, and thus this repair pathway can be utilized to treat those diseases for which mutating a genetic element may result in clinical benefit [9].

## HDR-mediated genome editing

### Site-specific gene correction

**Figure 1.**
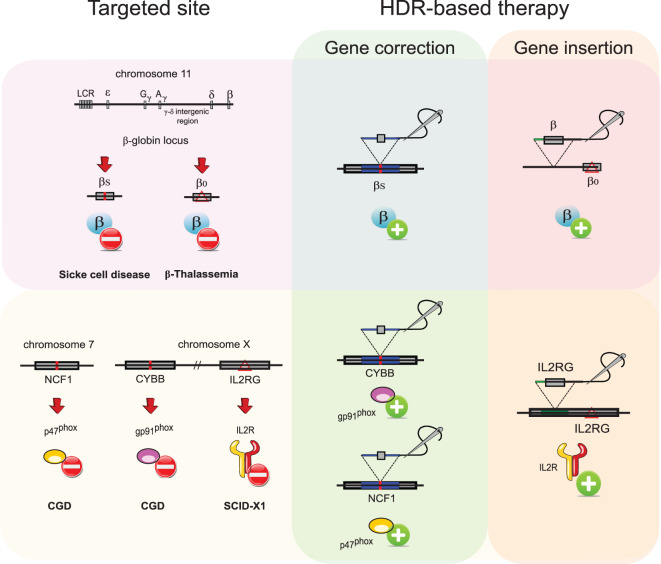
HDR-mediated genome editing. HDR is a cellular pathway that can be exploited to modify genomic sequences via site-specific gene correction of mutations (green box) or insertion of a correct gene (orange box). Mutations in the β-globin locus cause SCD and/or β-thalassemia. Abnormal β-globin (βs phenotype) is caused by an A-to-T point mutation; therefore, site-specific correction of the mutation could revert the disease phenotype. The same holds true for some forms of CGD, where the presence of mutation hotspots in the *CYBB* and *NFC1A* genes make this disease amenable to site-specific correction through gene editing. β-globin loss (β0 phenotype) in β-thalassemia or absence of the common gamma chain of IL-2 receptor in SCID-X1 is caused by mutations scattered around the respective genes. Healthy phenotypes in β-thalassemia (red box) or SCID-X1 (orange box) patients can be reconstituted via insertion of a correct gene in its own endogenous locus or in a ‘safe harbour’ genomic region.

Site-specific correction of disease-causing mutations represents the most straightforward approach to repair a faulty gene responsible for a monogenic disorder. *In situ* correction has the advantage of preserving endogenous regulatory regions and physiological gene expression, which would be particularly advantageous when targeting tightly regulated genes. Conditions for which a single or predominant mutation underlies the disease would seem to be the most amenable to this approach (Figure 1). An example is sickle cell disease (SCD). SCD is an autosomal recessive disorder affecting millions of people worldwide and is caused by an A-to-T point mutation in the sixth codon of the β-globin gene (HBB), resulting in the production of a defective globin that confers a hook/sickle shape to red blood cells [10]. ZFNs and CRISPR/Cas9 together with a donor template delivered via integration-defective lentiviral vectors (IDLV) or single-stranded DNA oligonucleotides (ssODN) have been extensively used to correct the SCD-causing mutation in different cell types [11–14]. In clinically relevant cells such as HSPCs, ZFN-mediated correction of the SCD mutation was achieved in up to 40% of the cells [15–17]. However, the frequency of correction dropped when cells were transplanted into immunodeficient mice, with only ∼0.2–2.3% of engrafted long-term repopulating HSPCs harbouring a corrected copy of the HBB gene at more than 12 weeks after transplantation, thus achieving rates of editing far below the level of therapeutic relevance. To overcome this limitation, Dever et al. deployed a strategy that allows the enrichment of edited HSPCs, by including a selectable marker into the HDR donor cassette delivered via a serotype 6 adeno-associated viral vectors (AAV6). Using this approach, the authors showed that >97% of cells engrafted into immunodeficient mice were gene targeted, a significant increase compared with mice transplanted with unselected gene-edited HSPCs (∼3%). Despite the increase in HDR rate, selection of targeted HSPCs before transplantation yielded an overall lower engraftment rate and recovery of fewer cells compared with standard protocols, indicating that further improvements in the selection technique and cell culture conditions are required before translating this strategy into the clinics.

Site-specific genome editing has also been attempted by the group of Harry Malech for the treatment of chronic granulomatous disease (CGD). CGD is a life-threatening disease caused by mutations in any of the five subunits (gp91^phox^, p22^phox^, p40, p47^phox^, p67^phox^) that comprise the phagocyte nicotinamide adenine dinucleotide phosphate oxidase (NADPH) complex. Apart from gp91^phox^, which is encoded by the CYBB gene located on the X chromosome, the remaining mutated subunits lead to an autosomal recessive form of the disease. While mutations are scattered across the genes for all other CGD patient phox genes, >80% of p47^phox^-CGD patients are homozygous for a two-nucleotide deletion in exon 2 of the NCF1 gene, resulting in a codon frameshift and abrogation of p47^phox^ expression [18]. Interestingly, the same mutation is found in the *NCF1* pseudogenes *NCF1B* and *NCF1C*. By delivering ZFNs and an AAV6 containing a correct exon 2 sequence as a template for HDR, Merling et al. replaced the mutated exon in the NCF1 locus as well as in its pseudogenes, restoring oxidase function in 6% of myeloid cells differentiated from patient's derived p47-CGD HSPCs and showing, for the first time, that rescue of a pseudogene function can correct a monogenic disorder [19]. In parallel, De Ravin et al. developed a CRISPR–Cas9 system to repair a point mutation in the CYBB gene, which is responsible for 6% of the cases of X-linked CGD (X-CGD), using a ssODN as a template to promote HDR-mediated gene repair. Seamless repair of the missense mutation restored gp91^phox^ protein expression and function in X-CGD HSPC-derived myeloid cells, and the amount of corrected cells decreased by <50% after transplant into immunodeficient mice [20].

### Site-specific gene insertion

One potential issue with site-specific gene correction is that the majority of genetic diseases are caused by mutations spanning across the genes, thus requiring tailoring of gene editing reagents for each individual patient. A more universal and attractive strategy would be to target an entire gene cassette to the desired locus so that, once integrated, the transgene would functionally correct all disease-causing mutations. To this aim, a whole expression cassette, including regulatory elements, can be inserted into a ‘safe harbour’, defined as a genomic region that is able to accommodate the expression of newly integrated DNA without adverse effects on the host cell or organism [21], such as the adeno-associated virus integration site 1 (AAVS1) locus. Once a set of safe harbour site-specific nucleases has been designed, the same toolbox could be potentially used to treat many different genetic disorders. Alternatively, it is possible to knock in the functional transgene into its own locus, allowing expression of the gene through its own endogenous regulatory elements. Although this strategy is specific for each single gene/disease, it could be particularly amenable for conditions in which endogenous gene regulation is essential (Figure 1).

Pioneering work by Urnov et al. showed that it is possible to correct a mutation responsible for the X-linked severe combined immune deficiency (SCID-X1) in primary cells using ZFNs directed against the IL-2 receptor common gamma-chain gene (IL2RG). By designing a donor template DNA carrying an exon 5 fragment of IL2RG, the authors reported up to 5% of HDR-mediated editing in primary T cells, paving the way for potential gene correction applications, aimed to treat SCID-X1 [22]. However, the modest rates of editing achieved in primary cells highlighted the limitation of plasmid transfection to deliver the nucleases and the HDR template, in particular when applied to cells sensitive to genetic manipulations such as HSPCs. Lombardi and colleagues first addressed this issue and managed to successfully insert a donor cassette downstream of the IL2RG promoter or in the safe harbour CCR5 locus, using an IDLV to deliver ZFNs and the HDR template to lymphoblastoid primary cells, embryonic stem cells and HSPCs. While this represented an improvement of the editing protocol for primary cells, knock-in rates, especially in therapeutically relevant HSPCs, were extremely low [23]. To overcome the poor integration level observed in repopulating stem cells, the same group then optimized various culture conditions, timing and delivery route of the reagents to enhance the insertion of an IL2RG cDNA cassette into the AAVS1 or the *IL2RG* locus in HSPCs derived from healthy or SCID-X1 donors, achieving up to 11% of gene targeting [24]. More recently, two preclinical studies have shown significant advancement in the correction of the IL2RG locus, by knocking in a full-length *IL2RG* cDNA delivered by an AAV6 donor vector using either the ZFN or CRISPR/Cas9 system [25,26]. Both groups were able to reach therapeutic levels of IL2RG expression in wild-type and SCID-X1 HSPCs, as well as in the more primitive population of HSCs, with *in vitro* and *in vivo* rescue of multi-lineage developmental potential from corrected cells. SCID-X1 represents an ideal target for proof-of-concept gene editing studies, as the tremendous selective advantage that functionally corrected cells have over mutated ones in a SCID setting [27] can compensate for the relatively low rate of HDR-mediated correction in HSPCs. Additional protocol optimization may be required to increase the percentage of gene correction in long-term repopulating stem cells to revert the disease phenotype in blood disorders where such a strong selective advantage is missing.

## NHEJ-mediated genome editing

Although gene correction might seem the most immediate approach to therapeutic genome editing, the first clinical trial using targeted nucleases in human patients has relied on NHEJ-based genetic disruption. One advantage of this strategy is that NHEJ tends to be a more active repair pathway compared with HDR, particularly in quiescent stem cells [28]. Another benefit of NHEJ over HDR is that it relies solely on targeting nucleases with no need for designing and producing a donor template, thus making the whole process less laborious and more efficient for a potential clinical application. The first-in-human genome editing trial (NCT01044654) used ZFNs in autologous T cells to target the HIV co-receptor CCR5 [29], with the aim to mimic naturally occurring mutations that abolish its expression and engender resistance to HIV infection [30]. The clinical outcome indicated that this NHEJ-based approach was safe for patients and able to confer a survival advantage *in vivo* against HIV. Preclinical *in vitro* and *in vivo* studies demonstrated the feasibility and safety of ZFN-based CCR5 disruption also in HSPCs, providing a potentially life-long treatment to HIV-infected patients [31]. The technology is currently being evaluated in an ongoing clinical trial (NCT02500849) as a collaboration between City of Hope Medical Center and Sangamo Therapeutics.

**Figure 2.**
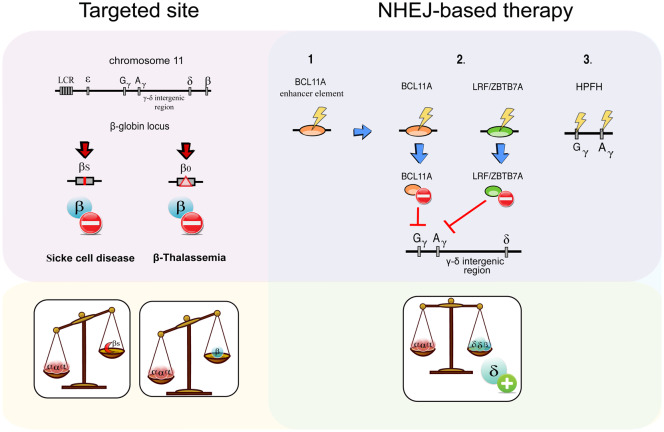
NHEJ-mediated genome editing. Genome editing can re-equilibrate the lost balance in the haemoglobin structure caused by the presence of defective subunits (pink and yellow boxes). The excess of one kind of haemoglobin chain — either α- or β-globin — can be reduced via NHEJ gene disruption (green box). Potential target sites to restore β-globin expression using an NHEJ-based approach are fetal haemoglobin (HbF) silencers (2, purple box), genomic regions regulating the expression of these silencers (1, purple box) or sites that can reproduce the HPFH phenotype (3, purple box).

The indels generated by NHEJ repair may be useful for disrupting not only coding sequences but also non-coding regulatory elements. For example, mutation of the erythroid-specific enhancer of BCL11A has been shown to increase fetal haemoglobin (HbF) expression and treat haemoglobinopathies. From the observations of new-born babies with β-globin defects [32], it is clear that by maintaining HbF expression it is possible to reduce or totally abolish the symptoms related to SCD or β-thalassemia [33–35]. BCL11A plays a crucial role in HbF transcriptional repression [36,37], in accordance with the rescue of therapeutically relevant levels of HbF expression in BCL11A knock-out murine models [38,39]. By using different gene editing platforms, many groups have shown that knock-out of BCL11A erythroid-specific enhancer restores high HbF levels, without compromising cell viability or function [38,40,41] (Figure 2). Preclinical studies made by Sangamo Therapeutics using ZFNs to target this region have demonstrated the therapeutic and clinical potential of the approach, obtaining engraftment of modified long-term HSCs *in vivo* and persisting production of high levels of HbF in animals [42–44]. At the beginning of the year, a Phase 1/2 clinical trial has started to evaluate the safety of their product in six β-thalassemia patients (NCT03432364); in parallel, CRISPR Therapeutics and Vertex Pharmaceuticals have opened a Phase 1/2 clinical trial on twelve SCD-affected subjects to test the safety of their CRISPR–Cas9 platform targeting the BCL11A enhancer in HSPCs (NCT03745287).

Apart from BCL11A, several other modulators of HBB expression have been identified as potential targets of NHEJ-based gene disruption approaches (Figure 2). In particular, it has been shown that CRISPR/Cas9-mediated knock-out of the lymphoma/leukemia-related factor (LRF), an HbF silencer [45], can increase more than five times the level of HbF expression. However, the involvement of LRF in other haematopoietic functions may impair the translation of this approach into the clinic and therefore it requires further investigation [46]. The presence of high levels of HbF in patients affected by the hereditary persistence of fetal haemoglobin (HPFH) benign condition has led several research groups to focus on genome editing strategies that could reproduce large deletions in the β-globin gene cluster and mutations in the γ-globin promoter region [47–49]. It has been shown that the disruption of HGB1 and HGB2 (γ-globin) gene promoter region inhibits the repression of HbF mediated by BCL11A or LRF reversing the globin switching [50,51]. Furthermore, natural HPFH-associated deletions and point mutations have been precisely generated *in vitro* in HSPCs through a CRISPR/Cas9 multiplex strategy [52,53], or a microhomology-mediated end joining microdeletion approach [54]. However, the reactivation of HbF expression at clinically beneficial levels has not been achieved so far, leaving open questions regarding the efficacy [55] and potential side effects *in vivo* [55] of the γ-globin silencer as a therapeutic target for gene editing.

## Epigenome and base editing

The great versatility and flexibility of the CRISPR/Cas9 platform and the ability of Cas9 to bind DNA independently from its nuclease activity led to the development of novel tools that could overcome some of the limitations of current gene editing approaches. Indeed, a ‘dead’ catalytically inactive Cas9 endonuclease (dCas9) has been engineered to tether different enzymatic activities to specific DNA sequences for a variety of applications, including transcriptional regulation and epigenetic modification.

Epigenetic marks and effectors have a crucial impact on chromatin organization and gene expression over time. For example, the effects of these modifications are clearly visible in the γ-to-β haemoglobin switching. Epigenome manipulation of the β-globin gene cluster, such as artificially driven changes that block epigenetic HbF gene silencing, offers a challenging alternative to the permanent alteration of the coding sequence that is achieved with ‘traditional’ genome engineering. Epigenome modifiers, composed of the LSD1 histone demethylase and ZFN [56,57] or TAL effector domains [58], were developed to target the globin locus control region (LCR), force the β-globin looping and restore HbF expression. One of these studies has shown that using this approach it is possible to induce a ∼2.5-fold increment in γ-globin expression in different donors [57]. Because LSD1 is essential for erythroid differentiation [59], the long-range precise control of the chromatin structure offers a potentially safer option for therapeutic purposes compared with the LSD1 knock-down or knock-out approach. Other potential epigenetic targets of interest for haemoglobinopathies are represented by the genes involved in the β-globin looping interactions such as methyl cytosine-binding domain proteins and the Mi2β chromatin remodelling ATPase [60,61], whose manipulation could be used to increase γ-globin gene expression without impairing erythroid differentiation.

As discussed above, the efficiency of HDR is very low due to the higher amount of DSBs being preferentially repaired by NHEJ. To introduce point mutations without using HDR, different platforms based on dCas9 fused to DNA deaminases have been developed for base editing technologies. Since 2016, four generations of base editors (BEs) have been developed from apolipoprotein B mRNA-editing enzyme catalytic polypeptide-like (APOBECs) or activation-induced cytidine deaminases combined with either the CRISPR–Cas9 or the CRISPR–Cpf1 system (reviewed in [62]). BEs could be therapeutically relevant to correct disease-causing point mutations or to induce site-specific mutations to disrupt genomic sequences. Although still in their early stage, few reports have shown the applicability of such technologies to treat haemoglobinopathies. Liu et al. [63] showed that plasmid delivery of an adenine BE was able to install a mutation known to confer HPFH and enable HbF production in HEK293T cells. Other two studies reported correction of a mutant HBB allele in β-thalassemia patient-derived erythroid precursors [64] and primary fibroblasts [65]. Despite being promising tools, BEs activity and editing efficiency in primary cells must be improved before moving from bench to clinic.

## Challenges of therapeutic genome editing

Site-specific genome editing has transformed the research field of biology and medicine. Not only can this technique allow the functional study of a particular gene, but it is also amenable to correction of a disease-associated mutation. Despite this exciting prospect, there remain major barriers associated with the editing-based therapeutic treatment of blood disorders that must be addressed to advance clinical applications that rely on genome editing. Some of these challenges include (1) the delivery of the editing machinery *ex vivo* and *in vivo*, (2) the ability to preserve the stemness and achieve high levels of engraftment of HSCs *in vivo*, (3) the identification and reduction in genome-wide off-target effects induced by the nucleases.

The main objective when delivering gene editing reagents is to promote a hit-and-run activity of the nucleases, to allow for the generation of DSBs in a short period of time, while limiting toxicity and off-target activity. For HDR-based gene addition approaches, it is also necessary to deliver the DNA donor template in a transient way, to avoid unwanted integration into non-specific regions of the genome. For decades, various delivery methods, such as electroporation, nanoparticles and viral vectors, have been used to introduce the editing reagents into cells for *ex vivo* or *in vivo* approaches. For *ex vivo* applications, transfection of plasmid DNA is nowadays the least used technique to target primary cells, as reports indicate induction of high toxicity, off-target insertion and host immune response [66,67]. These unwanted effects are observed minimally when delivering nucleases as mRNA or, in the case of Cas9 and the gRNA, in the form of a ribonucleoprotein complex [16,68–70]. Unlike electroporation, viral vectors, such as recombinant AAV and IDLV, have been applied in both *ex vivo* and *in vivo* preclinical studies. In addition to the delivery of editing reagents, their non-integrative properties allow them to be used to provide the donor template for HDR-based gene correction. The relatively low toxicity and non-integrative nature of AAV, together with its ability to achieve high recombination frequencies with small homology regions, have made them desirable genetic tools for efficient human HSPC gene editing [25,71–73]. Despite being safe, one of the drawbacks of AAV is their limited cargo capacity of 4.5 kb that restricts the expression of large transgenes, although it has been recently reported the integration of a cassette with up to 6.5 kb in size using a multiplexing strategy [74]. Delivery through lipid- and gold-based nanoparticles is considered safer *in vivo* compared with the use of viral vectors, but the targeting efficiency is still below therapeutic levels [75,76].

As outlined at the beginning of this review, long-term repopulating HSCs are the ideal target for gene editing of various types of inherited haematological conditions [16]. The therapeutic benefit of gene-corrected HSCs depends on their capacity to engraft and provide long-term production of healthy blood lineage progenitors while maintaining renewable stem cells in transplanted patients. One of the hurdles associated with HSC editing is the low rate of HDR than can be achieved, mainly due to the fact that NHEJ is preferentially utilized to correct DSBs in non-dividing cells. To overcome this problem, various groups have tried either pharmacological or genetic inhibition of NHEJ [77–80], strategies to increase the rates of HDR [81,82] and synchronization of the cell cycle [83], as well as optimization of delivery conditions to enhance knock-in efficiencies. Moreover, strategies to *in vitro* expand primitive HSC while preserving their stem cell qualities have been put in place, with the final aim to advance efforts at HSC modification, engraftment and long-term repopulation in *in vivo* xenotransplantation models [24,72,84].

The intrinsic specificity of the editing machinery permits researchers to modify their chosen gene of interest at a particular locus; however, unintended off-target cleavage at different genomic sites might occur. Being a permanent genetic modification, off-target cleavage could introduce unwanted mutations which may ultimately lead to cancer, posing a huge risk for clinical therapeutic applications involving engineered nucleases. Therefore, evaluation of off-target mutagenesis is an important preclinical criterion that must be taken into account before starting clinical studies. Several off-target detection methods have been developed to assess the safety of gene editing reagents. Early developed approaches have used computational predictions to identify a limited set of genomic regions that show homology with the target site, which can be then examined for a-specific cleavage by deep sequencing [85]. More recently, unbiased methods have been proposed that allow for genome-wide assessment of off-target mutagenesis, and in certain cases can also identify gross chromosomal rearrangements [86–89]. However, these methods do not always reliably pinpoint all potential off-targets and often show a limited sensitivity. To complicate the scenario, one must take into account the baseline level of mutagenesis that exists in normal somatic tissues or that can be caused by cell expansion during the manufacturing process. Dosage and expression pattern of the nucleases, as well as cell number, cell type and features of the genomic target site, may also affect editing specificity. It is difficult to predict the impact that modifications at different genomic sites may have on cell fitness, considering that most likely the vast majority of off-target edits would be functionally neutral. Interpreting the effects of genomic perturbations is challenging and requires the identification and development of functional readouts of safety that must be tailored to the therapeutic cell type of interest. There have been several efforts to increase the specificity of genome-editing systems, such as the development of shorter gRNAs [85], nickase mutants of Cas9, Cas9–FokI fusion protein [90,91], and high-fidelity Cas9 variants [69,92]. The continued improvements of the efficiency and safety of these reagents will be essential to ensure the success of gene editing applications to treat genetic haematological disorders.

## Conclusion

During the last few years, gene editing has emerged as a powerful tool for genomic functional studies and therapeutic correction of monogenic disease. Gene editing platforms, such as CRISPR/Cas9 and TALEN, have shown great promise in proof-of-principle preclinical studies to treat haematological disorders, and clinical trials using these tools are now underway. Nonetheless, there remain important challenges that need to be addressed, such as the efficiency and specificity of the editing system, especially when considering targeting of primary stem cells.

## Summary

- Genome editing is a rapidly evolving technology that has particular relevance for the treatment of haematological disorders and infectious diseases.
- Blood disorders can be tackled by taking advantage of two main endogenous cell repair mechanisms — NHEJ and HDR — which lead to either disruption of genes and regulatory elements or insertion of a correct gene in a site-specific fashion.
- Despite the tremendous progress, several issues need to be addressed to make genome editing a safer and more effective therapeutic tool.

## Abbreviations

AAVS1: adeno-associated virus integration site 1
BEs: base editors
CGD: chronic granulomatous disease
DSBs: double-strand breaks
HDR: homology-directed repair
HPFH: hereditary persistence of fetal haemoglobin
HR: homologous recombination
HSCs: haematopoietic stem cells
HSCT: haematopoietic stem cell transplantation
HSPCs: haematopoietic stem and progenitor cell
IDLV: integration-defective lentiviral vectors
IL2RG: IL-2 receptor common gamma-chain gene
LCR: locus control region
LRF: lymphoma/leukemia-related factor
NHEJ: non-homologous end joining
SCD: sickle cell disease
SCID: severe combined immunodeficiency disorders
SCID-X1: X-linked severe combined immune deficiency
ssODN: single-stranded DNA oligonucleotides
TALENs: transcription activator-like effector nucleases
X-CGD: X-linked CGD
ZFNs: zinc-finger nucleases

## References

[1] Kim H. and Kim J.-S. (2014) A guide to genome engineering with programmable nucleases. Nat. Rev. Genet. 15, 321–334 10.1038/nrg3686 24690881

[2] Liang L., Deng L., Chen Y., Li G.C., Shao C. and Tischfield J.A. (2005) Modulation of DNA end joining by nuclear proteins. J. Biol. Chem. 280, 31442–31449 10.1074/jbc.M503776200 16012167

[3] Lieber M.R., Ma Y., Pannicke U. and Schwarz K. (2003) Mechanism and regulation of human non-homologous DNA end-joining. Nat. Rev. Mol. Cell Biol. 4, 712–720 10.1038/nrm1202 14506474

[4] Locatelli F., Kabbara N., Ruggeri A., Ghavamzadeh A., Roberts I., Li C.K. et al. (2013) Outcome of patients with hemoglobinopathies given either cord blood or bone marrow transplantation from an HLA-identical sibling. Blood 122, 1072–1078 10.1182/blood-2013-03-489112 23692854

[5] Pai S.-Y., Logan B.R., Griffith L.M., Buckley R.H., Parrott R.E., Dvorak C.C. et al. (2014) Transplantation outcomes for severe combined immunodeficiency, 2000–2009. N. Engl. J. Med. 371, 434–446 10.1056/NEJMoa1401177 25075835 PMC4183064

[6] Thrasher A.J. and Williams D.A. (2017) Evolving gene therapy in primary immunodeficiency. Mol. Ther. 25, 1132–1141 10.1016/j.ymthe.2017.03.018 28366768 PMC5417846

[7] Biasco L., Rothe M., Büning H. and Schambach A. (2017) Analyzing the genotoxicity of retroviral vectors in hematopoietic cell gene therapy. Mol. Ther. Methods Clin. Dev. 8, 21–30 10.1016/j.omtm.2017.10.002 29159200 PMC5684499

[8] David R.M. and Doherty A.T. (2017) Viral vectors: the road to reducing genotoxicity. Toxicol. Sci. 155, 315–325 10.1093/toxsci/kfw220 27803388

[9] Porteus M.H. (2015) Towards a new era in medicine: therapeutic genome editing. Genome Biol. 16, 286 10.1186/s13059-015-0859-y 26694713 PMC4699361

[10] Ingram V. (1959) Abnormal human haemoglobins. III. The chemical difference between normal and sickle cell haemoglobins. Biochim. Biophys. Acta 36, 402–411 10.1016/0006-3002(59)90183-0 13852872

[11] Chang J.C., Ye L. and Kan Y.W. (2006) Correction of the sickle cell mutation in embryonic stem cells. Proc. Natl Acad. Sci. U.S.A. 103, 1036–1040 10.1073/pnas.0510177103 16407095 PMC1326143

[12] Zou J., Mali P., Huang X., Dowey S.N. and Cheng L. (2011) Site-specific gene correction of a point mutation in human iPS cells derived from an adult patient with sickle cell disease. Blood 118, 4599–4608 10.1182/blood-2011-02-335554 21881051 PMC3208277

[13] Sun N. and Zhao H. (2014) Seamless correction of the sickle cell disease mutation of the HBB gene in human induced pluripotent stem cells using TALENs. Biotechnol. Bioeng. 111, 1048–1053 10.1002/bit.25018 23928856

[14] Xie F., Ye L., Chang J.C., Beyer A.I., Wang J., Muench M.O. et al. (2014) Seamless gene correction of β-thalassemia mutations in patient-specific iPSCs using CRISPR/Cas9 and *piggyBac*. Genome Res. 24, 1526–1533 10.1101/gr.173427.114 25096406 PMC4158758

[15] Hoban M.D., Cost G.J., Mendel M.C., Romero Z., Kaufman M.L., Joglekar A.V. et al. (2015) Correction of the sickle cell disease mutation in human hematopoietic stem/progenitor cells. Blood 125, 2597–2604 10.1182/blood-2014-12-615948 25733580 PMC4408287

[16] Hoban M.D., Orkin S.H. and Bauer D.E. (2016) Genetic treatment of a molecular disorder: gene therapy approaches to sickle cell disease. Blood 127, 839–848 10.1182/blood-2015-09-618587 26758916 PMC4760089

[17] DeWitt M.A., Magis W., Bray N.L., Wang T., Berman J.R., Urbinati F. et al. (2016) Selection-free genome editing of the sickle mutation in human adult hematopoietic stem/progenitor cells. Sci. Transl. Med. 8, 360ra134 10.1126/scitranslmed.aaf9336 PMC5500303 27733558

[18] Casimir C.M., Bu-Ghanim H.N., Rodaway A.R., Bentley D.L., Rowe P. and Segal A.W. (1991) Autosomal recessive chronic granulomatous disease caused by deletion at a dinucleotide repeat. Proc. Natl Acad. Sci. U.S.A. 88, 2753–2757 10.1073/pnas.88.7.2753 2011585 PMC51317

[19] Merling R.K., Kuhns D.B., Sweeney C.L., Wu X., Burkett S., Chu J. et al. (2017) Gene-edited pseudogene resurrection corrects p47^phox^-deficient chronic granulomatous disease. Blood Adv. 1, 270–278 10.1182/bloodadvances.2016001214 29296942 PMC5727772

[20] De Ravin S.S., Li L., Wu X., Choi U., Allen C., Koontz S. et al. (2017) CRISPR-Cas9 gene repair of hematopoietic stem cells from patients with X-linked chronic granulomatous disease. Sci. Transl. Med. 9, eaah3480 10.1126/scitranslmed.aah3480 28077679

[21] Sadelain M., Papapetrou E.P. and Bushman F.D. (2011) Safe harbours for the integration of new DNA in the human genome. Nat. Rev. Cancer 12, 51–58 10.1038/nrc3179 22129804

[22] Urnov F.D., Miller J.C., Lee Y.-L., Beausejour C.M., Rock J.M., Augustus S. et al. (2005) Highly efficient endogenous human gene correction using designed zinc-finger nucleases. Nature 435, 646–651 10.1038/nature03556 15806097

[23] Lombardo A., Genovese P., Beausejour C.M., Colleoni S., Lee Y.-L., Kim K.A. et al. (2007) Gene editing in human stem cells using zinc finger nucleases and integrase-defective lentiviral vector delivery. Nat. Biotechnol. 25, 1298–1306 10.1038/nbt1353 17965707

[24] Genovese P., Schiroli G., Escobar G., Di Tomaso T., Firrito C., Calabria A. et al. (2014) Targeted genome editing in human repopulating haematopoietic stem cells. Nature 510, 235–240 10.1038/nature13420 24870228 PMC4082311

[25] Schiroli G., Ferrari S., Conway A., Jacob A., Capo V., Albano L. et al. (2017) Preclinical modeling highlights the therapeutic potential of hematopoietic stem cell gene editing for correction of SCID-X1. Sci. Transl. Med. 9, eaan0820 10.1126/scitranslmed.aan0820 29021165

[26] Pavel-Dinu M., Wiebking V., Dejene B.T., Srifa W., Mantri S., Nicolas C. et al. (2018) Gene correction for SCID-X1 in long-term hematopoietic stem cells. bioRxiv [cited 2019 Jan 16] 10.1101/397463 PMC6456568 30967552

[27] Bousso P., Wahn V., Douagi I., Horneff G., Pannetier C., Deist F.L. et al. (2000) Diversity, functionality, and stability of the T cell repertoire derived in vivo from a single human T cell precursor. Proc. Natl Acad. Sci. U.S.A. 97, 274–278 10.1073/pnas.97.1.274 10618408 PMC26653

[28] Mohrin M., Bourke E., Alexander D., Warr M.R., Barry-Holson K., Le Beau M.M. et al. (2010) Hematopoietic stem cell quiescence promotes error-prone DNA repair and mutagenesis. Cell Stem Cell. 7, 174–185 10.1016/j.stem.2010.06.014 20619762 PMC2924905

[29] Tebas P., Stein D., Tang W.W., Frank I., Wang S.Q., Lee G. et al. (2014) Gene editing of *CCR5* in autologous CD4T cells of persons infected with HIV. N. Engl. J. Med. 370, 901–910 10.1056/NEJMoa1300662 24597865 PMC4084652

[30] Hütter G., Nowak D., Mossner M., Ganepola S., Müssig A., Allers K. et al. (2009) Long-term control of HIV by CCR5 Delta32/Delta32 stem-cell transplantation. N. Engl. J. Med. 360, 692–698 10.1056/NEJMoa0802905 19213682

[31] DiGiusto D.L., Cannon P.M., Holmes M.C., Li L., Rao A., Wang J. et al. (2016) Preclinical development and qualification of ZFN-mediated CCR5 disruption in human hematopoietic stem/progenitor cells. Mol. Ther. Methods Clin. Dev. 3, 16067 10.1038/mtm.2016.67 27900346 PMC5102145

[32] Watson J. (1948) The significance of the paucity of sickle cells in newborn Negro infants. Am. J. Med. Sci. 215, 419–423 10.1097/00000441-194804000-00008 18107723

[33] Huisman T.H. (1979) Sickle cell anemia as a syndrome: a review of diagnostic features. Am. J. Hematol. 6, 173–184 10.1002/ajh.2830060210 382840

[34] Perrine R.P., Brown M.J., Clegg J.B., Weatherall D.J. and May A. (1972) Benign sickle-cell anæmia. Lancet 300, 1163–1167 10.1016/S0140-6736(72)92592-5 4117591

[35] Serjeant G.R. (2013) The natural history of sickle cell disease. Cold Spring Harb. Perspect. Med. 3, a011783 10.1101/cshperspect.a011783 23813607 PMC3784812

[36] Menzel S., Garner C., Gut I., Matsuda F., Yamaguchi M., Heath S. et al. (2007) A QTL influencing F cell production maps to a gene encoding a zinc-finger protein on chromosome 2p15. Nat. Genet. 39, 1197–1199 10.1038/ng2108 17767159

[37] Uda M., Galanello R., Sanna S., Lettre G., Sankaran V.G., Chen W. et al. (2008) Genome-wide association study shows BCL11A associated with persistent fetal hemoglobin and amelioration of the phenotype of β-thalassemia. Proc. Natl Acad. Sci. U.S.A. 105, 1620–1625 10.1073/pnas.0711566105 18245381 PMC2234194

[38] Bauer D.E., Kamran S.C., Lessard S., Xu J., Fujiwara Y., Lin C. et al. (2013) An erythroid enhancer of BCL11A subject to genetic variation determines fetal hemoglobin level. Science 342, 253–257 10.1126/science.1242088 24115442 PMC4018826

[39] Smith E.C., Luc S., Croney D.M., Woodworth M.B., Greig L.C., Fujiwara Y. et al. (2016) Strict in vivo specificity of the Bcl11a erythroid enhancer. Blood 128, 2338–2342 10.1182/blood-2016-08-736249 27707736 PMC5106112

[40] Canver M.C., Smith E.C., Sher F., Pinello L., Sanjana N.E., Shalem O. et al. (2015) BCL11A enhancer dissection by Cas9-mediated in situ saturating mutagenesis. Nature 527, 192–197 10.1038/nature15521 26375006 PMC4644101

[41] Vierstra J., Reik A., Chang K.-H., Stehling-Sun S., Zhou Y.-Y., Hinkley S.J. et al. (2015) Functional footprinting of regulatory DNA. Nat. Methods 12, 927–930 10.1038/nmeth.3554 26322838 PMC5381659

[42] Chang K.-H., Smith S.E., Sullivan T., Chen K., Zhou Q., West J.A. et al. (2017) Long-term engraftment and fetal globin induction upon *BCL11A* gene editing in bone-marrow-derived CD34^+^ hematopoietic stem and progenitor cells. Mol. Ther. Methods Clin. Dev. 4, 137–148 10.1016/j.omtm.2016.12.009 28344999 PMC5363298

[43] Ando D., Walters M.C., Chang K.-H., Reik A., Urnov F.D., Lee G. et al. (2015) Preclinical studies for the first hematopoietic stem cell (HSC) gene editing trial: phase 1 study of β-thalassemia with autologous transplantation of zinc finger nuclease-treated HSC to upregulate fetal hemoglobin. Mol. Ther. 23, S93–S94 10.1016/S1525-0016(16)33844-8

[44] Urnov F.D., Reik A., Vierstra J., Chang K.-H., Zhou Y., Mich A. et al. (2015) Clinical-scale genome editing of the human BCL11A erythroid enhancer for treatment of the hemoglobinopathies. Blood 126, 204

[45] Maeda T., Ito K., Merghoub T., Poliseno L., Hobbs R.M., Wang G. et al. (2009) LRF is an essential downstream target of GATA1 in erythroid development and regulates BIM-dependent apoptosis. Dev. Cell 17, 527–540 10.1016/j.devcel.2009.09.005 19853566 PMC3134301

[46] Lunardi A., Guarnerio J., Wang G., Maeda T. and Pandolfi P.P. (2013) Role of LRF/Pokemon in lineage fate decisions. Blood 121, 2845–2853 10.1182/blood-2012-11-292037 23396304 PMC3624932

[47] Sankaran V.G., Greisman H.A., Weatherall D.J., Groudine M. and Premawardhena A. (2011) A functional element necessary for fetal hemoglobin silencing. N. Engl. J. Med. 365, 807–814 10.1056/NEJMoa1103070 21879898 PMC3174767

[48] Bauer D.E., Kamran S.C. and Orkin S.H. (2012) Reawakening fetal hemoglobin: prospects for new therapies for the β-globin disorders. Blood 120, 2945–2953 10.1182/blood-2012-06-292078 22904296 PMC4467860

[49] Traxler E.A., Yao Y., Wang Y.-D., Woodard K.J., Kurita R., Nakamura Y. et al. (2016) A genome-editing strategy to treat β-hemoglobinopathies that recapitulates a mutation associated with a benign genetic condition. Nat. Med. 22, 987–990 10.1038/nm.4170 27525524 PMC5706766

[50] Liu N., Hargreaves V.V., Zhu Q., Kurland J.V., Hong J., Kim W. et al. (2018) Direct promoter repression by BCL11A controls the fetal to adult hemoglobin switch. Cell 173, 430–442.e17 10.1016/j.cell.2018.03.016 29606353 PMC5889339

[51] Martyn G.E., Wienert B., Yang L., Shah M., Norton L.J., Burdach J. et al. (2018) Natural regulatory mutations elevate the fetal globin gene via disruption of BCL11A or ZBTB7A binding. Nat. Genet. 50, 498–503 10.1038/s41588-018-0085-0 29610478

[52] Ye L., Wang J., Tan Y., Beyer A.I., Xie F., Muench M.O. et al. (2016) Genome editing using CRISPR-Cas9 to create the HPFH genotype in HSPCs: an approach for treating sickle cell disease and β-thalassemia. Proc. Natl Acad. Sci. U.S.A. 113, 10661–10665 10.1073/pnas.1612075113 27601644 PMC5035856

[53] Antoniani C., Meneghini V., Lattanzi A., Felix T., Romano O., Magrin E. et al. (2018) Induction of fetal hemoglobin synthesis by CRISPR/Cas9-mediated editing of the human β-globin locus. Blood 131, 1960–1973 10.1182/blood-2017-10-811505 29519807

[54] Traxler E., Yao Y., Li C., Grevet J., Huang P., Wright S. et al. (2015) Genome editing recreates hereditary persistence of fetal hemoglobin in primary human erythroblasts. Blood 126, 640 10.1182/blood-2015-03-635532 26084673 PMC4520879

[55] Chung J.E., Magis W., Vu J., Heo S.-J., Wartiovaara K., Walters M.C. et al.) CRISPR-Cas9 interrogation of a putative fetal globin repressor in human erythroid cells. PLoS ONE 14, e0208237 10.1371/journal.pone.0208237 PMC6333401 30645582

[56] Deng W., Lee J., Wang H., Miller J., Reik A., Gregory P.D. et al. (2012) Controlling long-range genomic interactions at a native locus by targeted tethering of a looping factor. Cell 149, 1233–1244 10.1016/j.cell.2012.03.051 22682246 PMC3372860

[57] Deng W., Rupon J.W., Krivega I., Breda L., Motta I., Jahn K.S. et al. (2014) Reactivation of developmentally silenced globin genes by forced chromatin looping. Cell 158, 849–860 10.1016/j.cell.2014.05.050 25126789 PMC4134511

[58] Mendenhall E.M., Williamson K.E., Reyon D., Zou J.Y., Ram O., Joung J.K. et al. (2013) Locus-specific editing of histone modifications at endogenous enhancers. Nat. Biotechnol. 31, 1133–1136 10.1038/nbt.2701 24013198 PMC3858395

[59] Xu J., Bauer D.E., Kerenyi M.A., Vo T.D., Hou S., Hsu Y.-J. et al. (2013) Corepressor-dependent silencing of fetal hemoglobin expression by BCL11A. Proc. Natl Acad. Sci. U.S.A. 110, 6518–6523 10.1073/pnas.1303976110 23576758 PMC3631619

[60] Kransdorf E.P., Wang S.Z., Zhu S.Z., Langston T.B., Rupon J.W. and Ginder G.D. (2006) MBD2 is a critical component of a methyl cytosine-binding protein complex isolated from primary erythroid cells. Blood 108, 2836–2845 10.1182/blood-2006-04-016394 16778143 PMC1895583

[61] Gnanapragasam M.N., Scarsdale J.N., Amaya M.L., Webb H.D., Desai M.A., Walavalkar N.M. et al. (2011) P66α–MBD2 coiled-coil interaction and recruitment of Mi-2 are critical for globin gene silencing by the MBD2–NuRD complex. Proc. Natl Acad. Sci. U.S.A. 108, 7487–7492 10.1073/pnas.1015341108 21490301 PMC3088629

[62] Rees H.A. and Liu D.R. (2018) Base editing: precision chemistry on the genome and transcriptome of living cells. Nat. Rev. Genet. 19, 770–788 10.1038/s41576-018-0059-1 30323312 PMC6535181

[63] Gaudelli N.M., Komor A.C., Rees H.A., Packer M.S., Badran A.H., Bryson D.I. et al. (2017) Programmable base editing of A•T to G•C in genomic DNA without DNA cleavage. Nature 551, 464–471 10.1038/nature24644 29160308 PMC5726555

[64] Gehrke J.M., Cervantes O., Clement M.K., Wu Y., Zeng J., Bauer D.E. et al. (2018) An APOBEC3A-Cas9 base editor with minimized bystander and off-target activities. Nat. Biotechnol. 36, 977–982 10.1038/nbt.4199 30059493 PMC6181770

[65] Liang P., Ding C., Sun H., Xie X., Xu Y., Zhang X. et al. (2017) Correction of β-thalassemia mutant by base editor in human embryos. Protein Cell 8, 811–822 10.1007/s13238-017-0475-6 28942539 PMC5676594

[66] Kim S., Kim D., Cho S.W., Kim J. and Kim J.-S. (2014) Highly efficient RNA-guided genome editing in human cells via delivery of purified Cas9 ribonucleoproteins. Genome Res. 24, 1012–1019 10.1101/gr.171322.113 24696461 PMC4032847

[67] Sun L., Wu J., Du F., Chen X. and Chen Z.J. (2013) Cyclic GMP-AMP synthase is a cytosolic DNA sensor that activates the type-I interferon pathway. Science 339, 786–791 10.1126/science.1232458 23258413 PMC3863629

[68] Hendel A., Bak R.O., Clark J.T., Kennedy A.B., Ryan D.E., Roy S. et al. (2015) Chemically modified guide RNAs enhance CRISPR-Cas genome editing in human primary cells. Nat. Biotechnol. 33, 985–989 10.1038/nbt.3290 26121415 PMC4729442

[69] Vakulskas C.A., Dever D.P., Rettig G.R., Turk R., Jacobi A.M., Collingwood M.A. et al. (2018) A high-fidelity Cas9 mutant delivered as a ribonucleoprotein complex enables efficient gene editing in human hematopoietic stem and progenitor cells. Nat. Med. 24, 1216–1224 10.1038/s41591-018-0137-0 30082871 PMC6107069

[70] Hoban M.D., Lumaquin D., Kuo C.Y., Romero Z., Long J., Ho M. et al. (2016) CRISPR/Cas9-mediated correction of the sickle mutation in human CD34^+^ cells. Mol. Ther. 24, 1561–1569 10.1038/mt.2016.148 27406980 PMC5113113

[71] Kuo C.Y., Long J.D., Campo-Fernandez B., de Oliveira S., Cooper A.R., Romero Z. et al. (2018) Site-specific gene editing of human hematopoietic stem cells for X-linked hyper-IgM syndrome. Cell Rep. 23, 2606–2616 10.1016/j.celrep.2018.04.103 29847792 PMC6181643

[72] Charlesworth C.T., Camarena J., Cromer M.K., Vaidyanathan S., Bak R.O., Carte J.M. et al. (2018) Priming human repopulating hematopoietic stem and progenitor cells for Cas9/sgRNA gene targeting. Mol. Ther. Nucleic Acids 12, 89–104 10.1016/j.omtn.2018.04.017 30195800 PMC6023838

[73] Dever D.P., Bak R.O., Reinisch A., Camarena J., Washington G., Nicolas C.E. et al. (2016) CRISPR/Cas9 β-globin gene targeting in human haematopoietic stem cells. Nature 539, 384–389 10.1038/nature20134 27820943 PMC5898607

[74] Bak R.O. and Porteus M.H. (2017) CRISPR-mediated integration of large gene cassettes using AAV donor vectors. Cell Rep. 20, 750–756 10.1016/j.celrep.2017.06.064 28723575 PMC5568673

[75] Finn J.D., Smith A.R., Patel M.C., Shaw L., Youniss M.R., van Heteren J. et al. (2018) A single administration of CRISPR/Cas9 lipid nanoparticles achieves robust and persistent in vivo genome editing. Cell Rep. 22, 2227–2235 10.1016/j.celrep.2018.02.014 29490262

[76] Lee K., Conboy M., Park H.M., Jiang F., Kim H.J., Dewitt M.A. et al. (2017) Nanoparticle delivery of Cas9 ribonucleoprotein and donor DNA in vivo induces homology-directed DNA repair. Nat. Biomed. Eng. 1, 889–901 10.1038/s41551-017-0137-2 29805845 PMC5968829

[77] Li J., Summerlin M., Nitiss K.C., Nitiss J.L. and Hanakahi L.A. (2017) TDP1 is required for efficient non-homologous end joining in human cells. DNA Repair 60, 40–49 10.1016/j.dnarep.2017.10.003 29078113

[78] Yu C., Liu Y., Ma T., Liu K., Xu S., Zhang Y. et al. (2015) Small molecules enhance CRISPR genome editing in pluripotent stem cells. Cell Stem Cell 16, 142–147 10.1016/j.stem.2015.01.003 25658371 PMC4461869

[79] Boitano A.E., Wang J., Romeo R., Bouchez L.C., Parker A.E., Sutton S.E. et al. (2010) Aryl hydrocarbon receptor antagonists promote the expansion of human hematopoietic stem cells. Science 329, 1345–1348 10.1126/science.1191536 20688981 PMC3033342

[80] North T.E., Goessling W., Walkley C.R., Lengerke C., Kopani K.R., Lord A.M. et al. (2007) Prostaglandin E2 regulates vertebrate haematopoietic stem cell homeostasis. Nature 447, 1007–1011 10.1038/nature05883 17581586 PMC2775137

[81] Li G., Zhang X., Zhong C., Mo J., Quan R., Yang J. et al. (2017) Small molecules enhance CRISPR/Cas9-mediated homology-directed genome editing in primary cells. Sci. Rep. 7, 8943 10.1038/s41598-017-09306-x 28827551 PMC5566437

[82] Song J., Yang D., Xu J., Zhu T., Chen Y.E. and Zhang J. (2016) RS-1 enhances CRISPR/Cas9- and TALEN-mediated knock-in efficiency. Nat. Commun. 7, 10548 10.1038/ncomms10548 26817820 PMC4738357

[83] Orthwein A., Noordermeer S.M., Wilson M.D., Landry S., Enchev R.I., Sherker A. et al. (2015) A mechanism for the suppression of homologous recombination in G1 cells. Nature 528, 422–426 10.1038/nature16142 26649820 PMC4880051

[84] Zonari E., Desantis G., Petrillo C., Boccalatte F.E., Lidonnici M.R., Kajaste-Rudnitski A. et al. (2017) Efficient ex vivo engineering and expansion of highly purified human hematopoietic stem and progenitor cell populations for gene therapy. Stem Cell Rep. 8, 977–990 10.1016/j.stemcr.2017.02.010 PMC5390102 28330619

[85] Fu Y., Foden J.A., Khayter C., Maeder M.L., Reyon D., Joung J.K. et al. (2013) High-frequency off-target mutagenesis induced by CRISPR-Cas nucleases in human cells. Nat. Biotechnol. 31, 822–826 10.1038/nbt.2623 23792628 PMC3773023

[86] Gabriel R., von Kalle C. and Schmidt M. (2015) Mapping the precision of genome editing. Nat. Biotechnol. 33, 150–152 10.1038/nbt.3142 25658281

[87] Frock R.L., Hu J., Meyers R.M., Ho Y.-J., Kii E. and Alt F.W. (2015) Genome-wide detection of DNA double-stranded breaks induced by engineered nucleases. Nat. Biotechnol. 33, 179–186 10.1038/nbt.3101 25503383 PMC4320661

[88] Tsai S.Q., Zheng Z., Nguyen N.T., Liebers M., Topkar V.V., Thapar V. et al. (2015) GUIDE-seq enables genome-wide profiling of off-target cleavage by CRISPR-Cas nucleases. Nat. Biotechnol. 33, 187–197 10.1038/nbt.3117 25513782 PMC4320685

[89] Kim D., Bae S., Park J., Kim E., Kim S., Yu H.R. et al. (2015) Digenome-seq: genome-wide profiling of CRISPR-Cas9 off-target effects in human cells. Nat. Methods 12, 237–243 10.1038/nmeth.3284 25664545

[90] Tsai S.Q., Wyvekens N., Khayter C., Foden J.A., Thapar V., Reyon D. et al. (2014) Dimeric CRISPR RNA-guided FokI nucleases for highly specific genome editing. Nat. Biotechnol. 32, 569–576 10.1038/nbt.2908 24770325 PMC4090141

[91] Mali P., Aach J., Stranges P.B., Esvelt K.M., Moosburner M., Kosuri S. et al. (2013) CAS9 transcriptional activators for target specificity screening and paired nickases for cooperative genome engineering. Nat. Biotechnol. 31, 833–838 10.1038/nbt.2675 23907171 PMC3818127

[92] Kleinstiver B.P., Pattanayak V., Prew M.S., Tsai S.Q., Nguyen N.T., Zheng Z. et al. (2016) High-fidelity CRISPR-Cas9 nucleases with no detectable genome-wide off-target effects. Nature 529, 490–495 10.1038/nature16526 26735016 PMC4851738

